# Sulforaphane and bladder cancer: a potential novel antitumor compound

**DOI:** 10.3389/fphar.2023.1254236

**Published:** 2023-09-15

**Authors:** Mingshun Zuo, Hongchuan Chen, Yuanjian Liao, Pingang He, Te Xu, Jiajia Tang, Neng Zhang

**Affiliations:** Department of Urology, The Affiliated Hospital of Zunyi Medical University, Zunyi, China

**Keywords:** bladder cancer, sulforaphane, MAPK, NF-κB, bladder outlet obstruction

## Abstract

Bladder cancer (BC) is a common form of urinary tract tumor, and its incidence is increasing annually. Unfortunately, an increasing number of newly diagnosed BC patients are found to have advanced or metastatic BC. Although current treatment options for BC are diverse and standardized, it is still challenging to achieve ideal curative results. However, Sulforaphane, an isothiocyanate present in cruciferous plants, has emerged as a promising anticancer agent that has shown significant efficacy against various cancers, including bladder cancer. Recent studies have demonstrated that Sulforaphane not only induces apoptosis and cell cycle arrest in BC cells, but also inhibits the growth, invasion, and metastasis of BC cells. Additionally, it can inhibit BC gluconeogenesis and demonstrate definite effects when combined with chemotherapeutic drugs/carcinogens. Sulforaphane has also been found to exert anticancer activity and inhibit bladder cancer stem cells by mediating multiple pathways in BC, including phosphatidylinositol-3-kinase (PI3K)/protein kinase B (Akt)/mammalian target of rapamycin (mTOR), mitogen-activated protein kinase (MAPK), nuclear factor kappa-B (NF-κB), nuclear factor (erythroid-derived 2)-like 2 (Nrf2), zonula occludens-1 (ZO-1)/beta-catenin (β-Catenin), miR-124/cytokines interleukin-6 receptor (IL-6R)/transcription 3 (STAT3). This article provides a comprehensive review of the current evidence and molecular mechanisms of Sulforaphane against BC. Furthermore, we explore the effects of Sulforaphane on potential risk factors for BC, such as bladder outlet obstruction, and investigate the possible targets of Sulforaphane against BC using network pharmacological analysis. This review is expected to provide a new theoretical basis for future research and the development of new drugs to treat BC.

## 1 Introduction

Bladder cancer (BC) is the most common malignancy of the urinary tract and ranks as the 10th most common malignant tumor globally. In 2020, there were 573,278 new diagnoses and 212,536 deaths due to BC (Sung et al., 2021). According to recent estimates, there will be 91,893 new cases of BC in China in 2022, resulting in 42,973 deaths, with a significantly higher incidence rate among males compared to females (Xia et al., 2022). Approximately 90% of BC cases are urothelial carcinomas, which are associated with high incidence and postoperative recurrence rates (Lenis et al., 2020). BC can be categorized according to tumor infiltration depth and staging as non-muscle invasive bladder cancer (NMIBC, 70%–75%, Ta, T1 and Tis) and muscle-invasive bladder cancer (MIBC, 25%–30%, T2-T4) (Seidl, 2020). However, the recurrence rate after the first surgery for NMIBC is approximately 70%, with 10%–20% progressing to MIBC (Kaufman et al., 2009). In MIBC patients, 25% of patients have lymph node metastases at diagnosis, and approximately 5% of patients develop distant metastases (Mokdad et al., 2017). In recent years, the proportion of newly diagnosed BC patients with advanced or metastatic disease has been rising, resulting in poor survival and prognosis. The 5-year survival rate for advanced or metastatic BC is only 15% (Mokdad et al., 2017).

Currently, a diverse range of standardized treatment options are available for BC. For all resectable non-metastatic MIBC patients, radical cystectomy and bilateral pelvic lymph node dissection are strongly recommended. However, MIBC patients still face the risk of postoperative recurrence, metastasis, and death (Patel et al., 2020; Slovacek et al., 2021). The first-line treatment for advanced or metastatic BC is platinum-based chemotherapy, which has a median overall survival (OS) of 15 months but is associated with intolerable side effects. The median OS of advanced or metastatic BC patients who are not eligible for platinum-based chemotherapy is only 9 months (Thomas and Sonpavde, 2022). Therefore, the treatment of BC still faces immense challenges, and there is an urgent need to discover effective new anti-tumor drugs. Interestingly, with the advancement of medical technology, plant extracts have shown significant potential for anti-cancer treatment, and there is an increasing interest in identifying specific plant compounds and their mechanisms of action.

Due to the complexity of bladder cancer, phytochemicals exhibit the following advantages in the treatment and prevention of BC: i) they are abundant in food sources, with proven efficacy, safety, tolerability, practicality, low cost, minimal side effects, and easy acceptance; ii) they share a common origin with medicines and foods: many phytochemicals with anticancer activity are derived from vegetables and fruits, and dietary habits are closely linked to cancer; iii) phytochemicals have a wide range of sources, both from extraction from plants and chemical synthesis; iv) most phytochemicals are metabolized and excreted in urine, allowing for more effective delivery to the bladder (Xia et al., 2021). Excitingly, the tissue uptake of sulforaphane is by far the highest, with urinary excretion ranging from 70% to 90% of the dose, with small individual differences and a tendency to accumulate in the bladder (Yagishita et al., 2019). Therefore, people are increasingly interested in paying attention to its therapeutic effects in BC.

**FIGURE 1:**
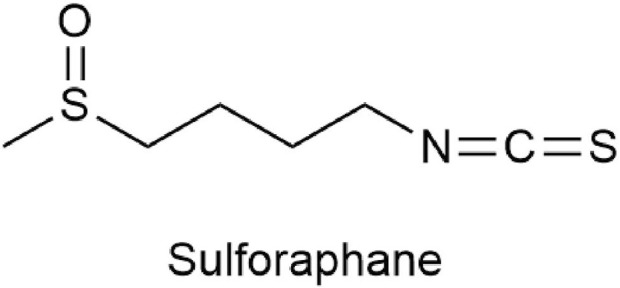
Chemical structure of sulforaphane.

Sulforaphane [SFN, C6H11S2NO,1-isothiocyanato-4-(methylsulfinyl)butane] (Figure 1) is an isothiocyanate found in cruciferous vegetables and is one of the hydrolytic products of glucosinolates from myrosinase in plants. It is abundant in cruciferous vegetables such as broccoli, cabbage, cauliflower, kale, and mustard greens and is currently the most effective plant-active substance discovered in vegetables for its anti-cancer effect (Vanduchova et al., 2019). Although SFN was isolated and identified in 1959, it did not gain people’s attention until 1992 when Prochaska et al. developed a method for screening fruits and vegetable extracts that can induce phase 2 enzymes (Prochaska et al., 1992). Current research has confirmed that sulforaphane not only has detoxifying (Alkharashi et al., 2019), antioxidant (Akbari and Namazian, 2020), anti-inflammatory (Al-Bakheit and Abu-Qatouseh, 2020), antibacterial (Deramaudt et al., 2020), immune-regulating (Mahn and Castillo, 2021), obesity-reducing (Çakır et al., 2022), cardiovascular disease-improving (Zhang et al., 2022), and diabetes-improving (Bose et al., 2020) effects but also has significant anti-cancer effects in many cancers, such as lung cancer (Wang et al., 2004), breast cancer (Fowke et al., 2003), colon cancer (Lin et al., 1998), and prostate cancer (Joseph et al., 2004). Due to the potent pharmacological effects of SFN, more and more studies are focusing on its impact on BC cells. Therefore, this article summarizes the effects and mechanisms of SFN on bladder cancer to clarify its therapeutic effects on BC.

## 2 Sulforaphane and its enantiomers and analogues

The absolute bioavailability of sulforaphane is about 80%, which is easily absorbed and eliminated, and its biological half-life is only a few hours. SFN is mainly metabolized through the mercapturic acid pathway *in vivo*. Once ingested, it is absorbed in the jejunum and passively diffused into the bloodstream. It first binds to the thiol of plasma proteins and enters the cell through the plasma membrane, then reacts with glutathione to form a conjugate, followed by a series of sequential transformations catalyzed by γ-glutamyltranspeptidase (γGT), cysteinylglycinase (CGase), and N-acetyltransferase (NAT). Finally, the conjugate is excreted by the transport protein and metabolized into mercapturic acid. Finally, these metabolites are transported to the kidneys and selectively transported to the bladder through urinary excretion (Mennicke et al., 1987; Chung et al., 1998). Therefore, due to the metabolic characteristics of SFN and its metabolites, it tends to accumulate in specific organs, especially the bladder (Bricker et al., 2014). After oral administration of SFN for 1.5, 6, and 24 h, the levels of bladder tissue in rats were 189.6 ± 49.0, 51.7 ± 10.9, and 6.9 ± 0.9 nmol/g, respectively. The maximum concentration in urine was about 50-fold higher than that in plasma (Veeranki et al., 2013; Bricker et al., 2014).

In addition, there are three main reasons why SFN can attract people’s interest: i) It naturally exists in a wide range of vegetables; ii) It is a highly effective phase 2 enzymes inducer; and iii) It is a single functional inducer (Prochaska and Talalay, 1988). However, Phase I enzymes may activate the carcinogen as the ultimate carcinogen, while the induction of cytoprotective phase 2 enzymes is sufficient for chemoprevention. SFN can induce phase 2 enzymes activity in the human body while inhibiting the production of phase I enzymes, ultimately eliminating carcinogenic substances and other harmful substances through various enzymatic systems (Tortorella et al., 2015). It is worth mentioning that SFN has the highest induction rate of phase 2 enzymes in the bladder among many organs (Veeranki et al., 2013), which has a high concentration in the bladder, and the bladder epithelium is the tissue with the most exposure to SFN and its metabolites, second only to gastric tissue (Zhang, 2004; Veeranki et al., 2013). Therefore, SFN is more effective than other target organs in preventing bladder cancer. It is of great significance to study the molecular mechanism of SFN against bladder cancer.

SFN consists of a basic structure consisting of β-D-thioglucose, an oxime sulfonate and a variable side chain composed of amino acids, including methionine, tryptophan, phenylalanine, or other amino acids. The side chain structure can contain alkyl, aryl, or heterocyclic groups, which determine the chemical, physical, and biological properties of isothiocyanates (Beal, 2009; Klomparens and Ding, 2019). Importantly, existing research data have confirmed that structural characteristics can determine the bioactivity of natural isothiocyanates, as even minor changes can have significant effects on their efficacy. Additionally, the oxidation state of sulfur can also alter the activity and potency of compounds, such as the fact that thioether I and sulfone II derivatives have lower activity than sulfoxide derivatives (sulforaphane) (Zhang et al., 1992; Rose et al., 2000; Juge et al., 2007). Therefore, based on the structural characteristics of SFN, there is great interest in exploring its enantiomers and analogs to obtain a chemically stable and biologically more active compound.

**FIGURE 2:**
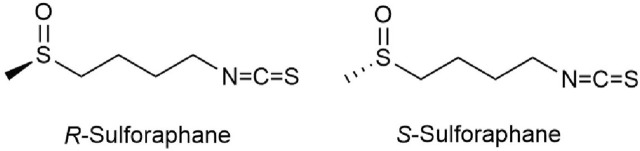
Chemical structure of two main sulforaphane enantiomers.

However, only a few studies have so far reported on the differences between SFN enantiomers and analogs. SFN exists in two forms of enantiomers, including natural (R)-SFN and unnatural (S)-SFN (Figure 2). Natural SFN exists as a single enantiomer with an RS absolute configuration (Vergara et al., 2008). In plants, natural SFN is mainly (R)-SFN, but depending on the plant species or location (flower, leaf, or stem), the content of (S)-SFN can account for 1.5%–41.8% of the total SFN (Okada et al., 2017). Abdull Razis et al. (Abdull Razis et al., 2011a) showed that (R)-SFN significantly upregulates the levels and activities of NAD(P)H: quinone oxidoreductase-1 (NQO1) and glutathione S-transferase (GST) in the liver and lungs of rats, while the activity of (S)-SFN is lower or ineffective. Further studies by Abdull Razis et al. demonstrate that glucuronosyl transferase and epoxide hydrolase are two major carcinogen-metabolizing enzyme systems, (R)-SFN and (S)-SFN can both enhance the activity of epoxide hydrolase in the rat liver, but the upregulation effect of (R)-SFN is stronger. In addition, (R)-SFN increases the expression and activity of glucuronosyl transferase, while (S)-SFN has the opposite effect (Abdull Razis et al., 2011b).

**FIGURE 3:**
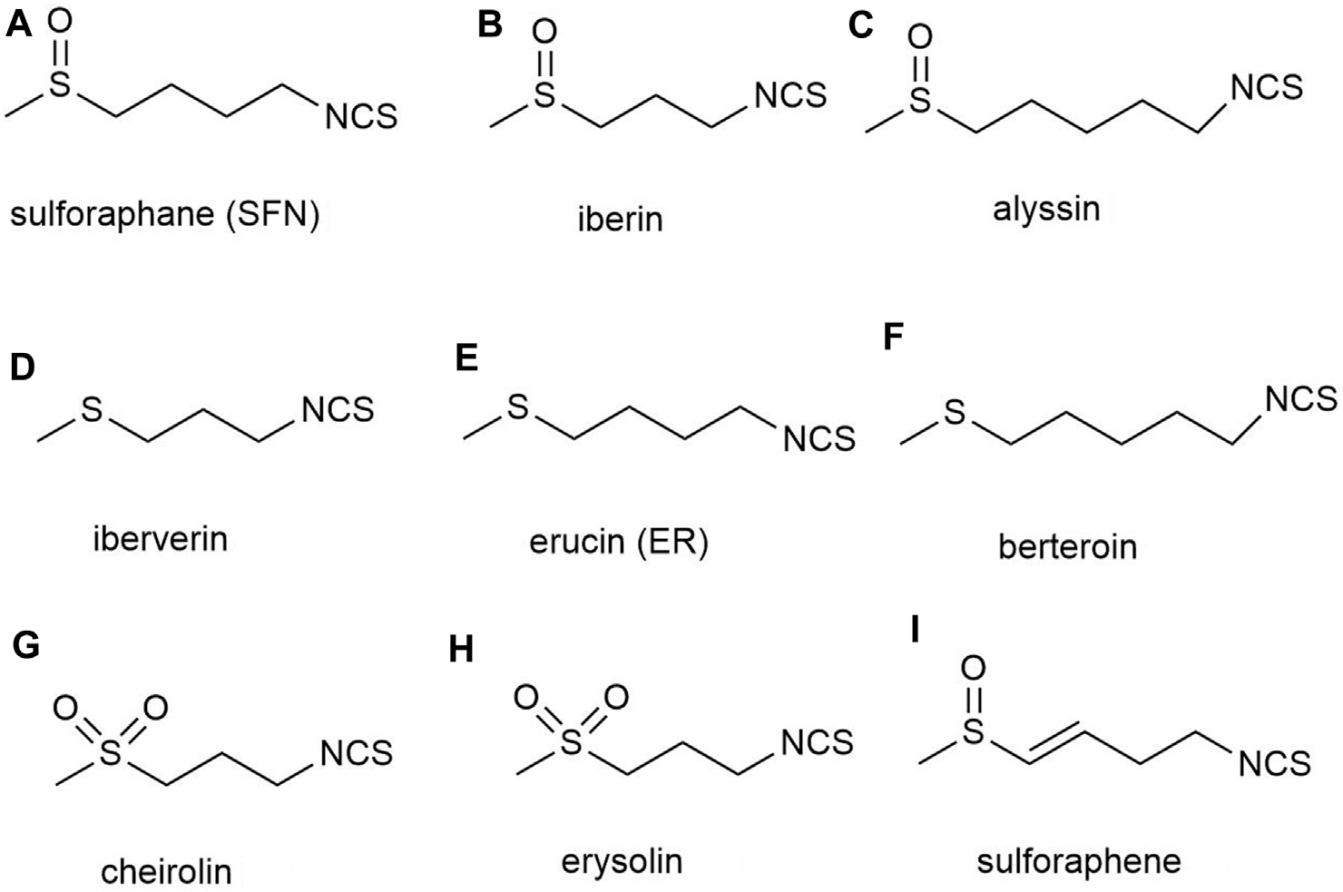
Structures of natural analogs of sulforaphane. **(A)** sulforaphane (SFN), **(B)** iberin, **(C)** alyssin, **(D)** iberverin, **(E)** erucin (ER), **(F)** berteroin, **(G)** cheirolin, **(H)** erysolin, **(I)** sulforaphene.

Although an increasing number of SFN analogues have been analyzed, no analogues with biological activity beyond natural SFN have been found (Elhalem et al., 2014; Kiełbasiński et al., 2014; Janczewski, 2022). The natural SFN analogues contain alkyl chains with 3–5 carbon atoms, and their II, IV, and VI sulfur atoms are in an oxidized state. However, these analogs include iberin b) and alyssin c) (Karrer et al., 1950) with a methylsulfonyl group, ibertin d) (Kjær et al., 1955a), erucin e) (Kjær and Gmelin, 1955) and berteroin f) (Kjær et al., 1955b) with a methylsulfonyl group, and cheirolin g) (Schneider, 1908) and erysolin h) (Schneider and Kaufmann, 1912) with a methylsulfonyl group. The α,β-unsaturated analog of sulforaphene i) is also known (Balenović et al., 1966) (Figure 3). However, researchers have not achieved satisfactory biological activity regardless of whether they converted the sulfoxide group in SFN structure into sulfones or sulfide in methyl mercaptan groups, or substituted sulfoxides with methylene or carbonyl groups, or changed the number of methylene units from 4 to 3 or 5. Although these analogs altered the rigidity of the methylene bridge and the structure of SFN, in all cases, the induction activity of phase 2 enzymes was not improved. And in certain instances, its activity was even decreased, demonstrating the superiority and importance of the natural SFN structure (Posner et al., 1994; Moriarty et al., 2006; Zhang and Tang, 2007).

## 3 Sulforaphane and BC

There is controversy surrounding epidemiological studies on the association between cruciferous vegetable intake and BC risk. According to the results of two case-control studies and one cohort study, there is a significant negative correlation between cruciferous vegetable intake and BC risk (Michaud et al., 1999; Castelao et al., 2004; Lin et al., 2009). However, some research has shown that there is no significant correlation between cruciferous vegetable intake and BC risk (Park et al., 2013; Nguyen et al., 2021; Yu et al., 2021). In a meta-analysis of a prospective study that included 1,503,016 participants, there was no significant dose-response relationship between cruciferous vegetable intake and BC (Yu et al., 2022). Additionally, in a meta-analysis of a systematic review of 41 studies and 303 observational studies that included 13,394,772 patients, the results showed no significant correlation between cruciferous vegetable intake and the health outcome of BC (Li Y. Z. et al., 2022). However, it is worth noting that despite the controversy surrounding the association between cruciferous vegetable intake and BC, an increasing number of studies are exploring the potential anti-BC properties of SFN.

Currently, many studies have reported the study of SFN and BC cell lines and animal models. Although the genetic heterogeneity and frequency of oncogenes and tumor suppressor genes vary among different types of BC, SFN has shown clear therapeutic effects on different subtypes of BC in both *in vivo* and *in vitro* experiments (Tables 1, 2). Some of the human bladder cancer cell lines that have been commonly used in in vitro experiments are RT4, T24, RT112, 5637, UM-UC-3, TCCSUP, J82, SW780 and others, some of which are subtyped as NMIBC, while others are subtyped as MIBC (Fujiyama et al., 2001; Arantes-Rodrigues et al., 2013; Conde et al., 2015; Liu et al., 2015). In these *in vitro* studies, researchers have treated various BC cell lines and animal models with specific concentrations of SFN to reveal the potential of SFN against BCa *in vitro*. This paper summarizes the existing *in vitro* studies of SFN and BC (Table 1).

**TABLE 1:** Sulforaphane and bladder cancer *in vitro*.

Compound	Dose	Experimental subjects	BC subtypes	IC50	Result/mechanism	Reference
1.SFN	SFN:1-40*μ*Μ, cisplatin:0.125–4 *μ*g/mL, gemcitabine:1.25-40*η*g/mL	RT4, RT112, T24, TCCSUP, resistant RT4, resistant TCCSUP	NMIBC, MIBC		SFN can inhibit sensitive and cisplatin- and gemcitabine-resistant cell growth and proliferation	Xie et al. (2022)
2.cisplatin						
3.gemcitabine						
SFN	0,10,20,40,80 μmol/L	SV-HUV-1, 5637, T24, J82, SW780, UM-UC-3	NMIBC, MIBC		SFN can inhibit the viability, migration and invasion of BC cells by inhibiting the expression of FAT1	Wang et al. (2020)
1.SFN	SFN/erucin:0,5,10,20 μM	RT4, J82, UMUC3, NHU	NMIBC, MIBC	1.NHU:not reached	SFN can block the cell cycle in G2/M phase, upregulate the expression of Caspase3/7 and PARP cleavage, and downregulate the expression of Survivin, EGFR and HER2/neu	Abbaoui et al. (2012)
2.erucin				2.RT4: 11.2 ± 0.3 μM		
				3.J82:7.7 ± 1.8 μM		
				4.UMUC3:5.66 ± 1.2 μM		
SFN	1,5,10,20 μmol/L	RT4, T24, UM-UC-3, HUVECs	NMIBC, MIBC		1.SFN inhibits the production of ATP by inhibiting glycolysis and mitochondrial oxidative phosphorylation in BC cells in a dose-dependent manner	Huang et al. (2022)
					2. SFN can reverse the dysregulation of glucose metabolism by inhibiting the AKT1-HK2 axis	
SFN	0,5,10,20 μM	RT4, RT112	NMIBC, MIBC		Sulforaphane inhibits glycolysis by down-regulating hypoxia-induced HIF-1α level and reducing HIF-1α nuclear translocation, thus inhibiting the proliferation of NMIBC.	Xia et al. (2019)
1.SFN	1.SFN:2.5 μM	RT112, UM-UC-3, TCCSUP	MIBC		1. The combination of everolimus and/or sulforaphane inhibits the growth and proliferation of BC cells by affecting cell cycle proteins and stopping the cell cycle	Justin et al. (2020a), Justin et al. (2020b)
2.everolimus	2.everolimus: 0.5ηM				2.The combination of everolimus and sulforaphane can inhibit the drug resistance of bladder cancer cells induced by long-term everolimus application	
3.SFN + everolimus	3. SFN:2.5 μM + everolimus: 0.5ηM					
SFN	0–160 μM	T24	MIBC		SFN inhibits the growth and migration of bladder cancer cells through GSH production and 2-phase enzyme expression mediated by Nrf2	He et al. (2021)
SFN	20 μM	5637	MIBC		SFN induces mitotic arrest and apoptosis of BC cells through ROS-dependent pathway	Park et al. (2014)
1.SFN	SFN:0–40 μM, TRAIL:0-500 ηg/mL	253J, EJ, 5637, T24, J82, HT1376	MIBC		SFN can upregulate ROS production and Nrf2 activity, and TRAIL significantly enhances SFN-mediated ROS production, which leads to the apoptosis of TRAIL-resistant bladder cancer cells	Jin et al. (2018)
2.TRAIL						
3.SFN + TRAIL						
1.SFN	SFN:0,2,4 μM, ABP:0.05,0.1,0.25,0.5 Mm, N-OH-AABP:0.05,0.1 mM	RT4	NMIBC		SFN inhibits ABP-induced DNA damage by activating Nrf2 signaling pathway in bladder cancer cells	Ding et al. (2010)
2.ABP						
3.N-OH-AABP						
SFN	0,5,10,15,20,30 μM	T24	MIBC		SFN plays an anti-tumor role by inducing apoptosis of bladder cancer cells through ROS-mediated intrinsic apoptosis pathway	Jo et al. (2014)
SFN	0,5,10,20 μM	T24	MIBC		SFN inhibits the invasion and metastasis of BC cells by inhibiting EMT process through Cox-2/MMP-2, 9/ZEB1 and Snail and miR-200c/ZEB1 pathways	Shan et al. (2013)
SFN	0,5,10,20 μM	T24	MIBC		P38 MAPK activation can promote SFN to regulate the expression of ARE-dependent enzymes and COX-2	Shan et al. (2010)
SFN	0,5,10,20 μM	RT4, J82, UM-UC-3	NMIBC, MIBC		SFN modulates the histone status in BC cells by regulating specific HDAC and HATs, and enhances phosphatase activity, thus reducing histone H1 phosphorylation	Abbaoui et al. (2017)
1.AZ	AZ, SFN, AZ + SFN:2.5,5,10,20,40,80 μM	HTB-9, RT112	NMIBC		combination AZ + SFN treatment inhibits BC cell growth, proliferation, cloning, cell cycle arrest, and induces apoptosis through caspase-3 and PARP activation	Islam et al. (2016a)
2.SFN						
3.AZ + SFN						
SFN	0–80 μM	T24	MIBC	24 h:26.9 ± 1.12 *μ*M	SFN blocks cell cycle progression in G0/G1 phase by upregulating p27 expression and induces early apoptosis to inhibit BC cells growth and progression	Shan et al. (2006)
				48 h:15.9 ± 0.76 μM		
SFN	0,5,10,20 μM	T24	MIBC	72 h:11.8 ± 0.7 μM	SFN downregulates COX-2 expression by activating p38 and inhibiting NF-kB-DNA binding activity in BC cells	Shan et al. (2009)
SFN	4–8 μM	NBT-II, Wild-type (Nrf2^+/+^) and Nrf2-deficient (Nrf2^−/−^) mouse embryonic fibroblasts	MIBC		SFN significantly inhibits the proliferation of BC cells by inducing the activities of GST and NQO1	Zhang et al. (2006)
SFN	0–40 μM	T24	MIBC		SFN may inhibit BC cell migration by activating autophagy, and it shows a bell-shaped dose response to BC cell growth and migration	Bao et al. (2014)
SFN	-	RT4, UM-UC-6, UM-UC-6/dox	NMIBC, MIBC		SFN effectively inhibits the growth of BC cells and doxorubicin-resistant derivative BC cells	Tang et al. (2006)
SFN	7.5–30 μM	UM-UC-3	MIBC		SFN can induce apoptosis of BC cells and damage mitochondria weakly	Tang and Zhang (2005)

IC50:Semi inhibitory concentration, FAT1: FAT, atypical cadherin 1, HUVECs: Human umbilical vein endothelial cell, PARP: poly (ADP-ribose) polymerase, EGFR: epidermal growth factor receptor, HER2: human epidermal growth factor receptor 2, HK2: hexokinase 2, HIF-1α:hypoxia induicible factor-1, alpha, GSH: glutathione, ROS: reactive oxygen species, TRAIL: tumor necrosis factor-related apoptosis inducing ligand, ABP: 4-aminobiphenyl, N-OH-AABP: N-hydroxy-N-acetyl-4-aminobiphenyl, EMT: epithelial-to-mesenchymal transition, COX-2: cyclooxygenase-2, MMP2: matrix metalloproteinase-2, ARE: antioxidant response element, HDAC: histone deacetylase, HAT: histone acetyltransferase, AZ: acetazolamide.

**TABLE 2:** *In vivo* study of sulforaphane and bladder cancer.

Animal models	BC subtypes	Dose	Delivery way	Tumor inhibition rate (%)	Result/mechanism	Reference
UMUC3 xenograft tumor model in mice	MIBC	295 μmol/kg	intragastric administration once daily for 2 weeks	42	SFN inhibits the occurrence, growth, and invasion of human bladder cancer cells	Abbaoui et al. (2012)
BBN-induced bladder tumor model in male C57BL/6 mice		2.5,5, 10 mg/kg	intragastric administration three times a week for 23 weeks		SFN reverses the dysregulation of glycolysis flux by inhibiting a variety of glucose metabolic enzymes	Huang et al. (2022)
Male wild-type C57BL/6 mice and Nrf2-deficient C57BL/6 mice		SFN:0,10,40	1.intragastric administration once daily for 5 days, and a single dose of ABP was injected intraperitoneally 3 h after the last SFN administration	10 *μ*mol/kg SFN:50%, 40 *μ*mol/kg SFN:61%	SFN inhibits ABP-induced DNA damage by activating Nrf2 signaling pathway in mouse bladder tissue	Ding et al. (2010)
		*μ*mol/kg ABP:50 mg/kg	2. intragastric administration once daily for 5 days			
UMUC3 xenograft tumor model in mice	MIBC	295 *μ*mol/kg	intragastric administration once daily for 2 weeks	10 ± 0.7	SFN regulates histone status through HDAC inhibition and phosphatase enhancement, reduces histone H1 phosphorylation and thereby inhibits BC progression	Abbaoui et al. (2017)
1.HTB-9 xenograft tumor model in mice	NMIBC, MIBC	1.AZ:20 mg/kg	intraperitoneal injection every day for 16 days		The combination of AZ and SFN can effectively target the malignant progression of bladder cancer by inhibiting tumor proliferation and growth, inducing apoptosis, and inhibiting the EMT process	Islam et al. (2016b)
2. RT112 xenograft tumor model in mice		2.SFN:40 mg/kg				
		3. AZ 20 mg/kg + SFN 40 mg/kg				
BBN-induced bladder tumor model in male C57BL/6 mice		2.5,5, 10 mg/kg	intragastric administration for 11–12 weeks		SFN can normalize intestinal flora, repair physiological damage of intestinal barrier, and reduce inflammation and immune response	He et al. (2018)
UMUC3 xenograft tumor model in mice	MIBC	12 mg/kg	intragastric administration twice a day for 5 weeks	63	SFN inhibits the growth of BC by anti-angiogenesis, immune regulation and induction of apoptosis	Wang and Shan (2012)

BBN, N-butyl-N-(4-hydroxybutyl) nitrosamine, ABP, 4-aminobiphenyl (ABP), HDAC, histone deacetylase, EMT, epithelial-to-mesenchymal transition.

Of course, many studies have also reported *in vivo* studies of SFN and BC animal models. In these *in vivo* studies, researchers constructed animal tumor models by subcutaneously or orthotopically implanting human bladder cancer cell lines into immunodeficient mice. Finally, researchers confirmed that SFN can effectively inhibit BC growth by treating animal models with different concentrations and methods of SFN. Similarly, this article summarizes the current *in vivo* research on SFN and BC (Table 2).

## 4 Sulforaphane and bladder outlet obstruction: bladder protection

Partial bladder outlet obstruction (pBOO) is a common chronic disease of the urinary system where blockages usually occur at the bottom and neck of the bladder, preventing normal urination. Currently, pBOO commonly occurs in benign prostatic hyperplasia (BPH), bladder neck spasm, urethral stricture, congenital urethral malformation, and bladder neck tumors, among which BPH is the most common cause of pBOO (MacDonald and McNicholas, 2003; Albisinni et al., 2016; Kai et al., 2020). BOO primarily induces progressive remodeling of bladder tissues through three consecutive stages: hypertrophy, compensation, and decompensation (Levin et al., 2004). In the short term, pBOO can temporarily improve bladder function by inducing bladder mucosal hyperplasia and detrusor smooth muscle hypertrophy. However, long-term BOO can cause loss of bladder smooth muscle, deposition of extracellular matrix, and degradation of neurons, leading to changes in the bladder’s tissue structure and decreased function (Kai et al., 2020; Lee et al., 2022).

Currently, some studies have confirmed that BOO may be related to the development of BC. Lin et al. confirmed that BOO induces bladder mucosal carcinogenesis by forming infrared spectral anomalies, through a study of changes in rabbit bladder mucosal conformation before and after BOO formation using Fourier transform infrared spectroscopy with attenuated total reflection techniques (Lin et al., 1994). In addition, a study analyzed the data of 66,782 patients in the National Health Insurance Research Database (NHIRD) found that adverse outcomes after treatment of BOO caused by elderly BPH, whether by drugs or surgery, were significantly associated with a higher incidence of BC (Lin et al., 2019). Furthermore, in rats treated with N-butyl-N-(4-hydroxybutyl) nitrosamine (BBN), it was found that pBOO-induced bladder hypertrophy, hyperplasia, angiogenesis, and hypoxia were significantly related to an increased incidence of BC, which would accelerate bladder carcinogenesis (Matsumoto et al., 2009). Therefore, actively treating BOO is of great significance for the prevention of BC.

Fortunately, SFN has shown definite therapeutic effects on BOO. In an *in vivo* study by Liu et al., SFN prolonged the voiding interval, increased bladder capacity, improved bladder compliance, and inhibited the increase of collagen fibers in BOO rats. In addition, they found that SFN could alleviate pBOO-induced bladder injury by activating the Nrf2-ARE pathway, increasing the activity of antioxidant enzymes such as superoxide dismutase (SOD), glutathione peroxidase (GSH-PX), and catalase (CAT) to reduce oxidative stress, and lowering the B cell lymphoma 2 associated X protein (Bax)/B cell lymphoma 2 (bcl-2) ratio to inhibit apoptosis (Liu et al., 2016). In Liu et al.'s subsequent *in vivo* study (Liu et al., 2019), through treating BOO rat models with 0.5 mg/kg/day of SFN, they found that bladder compliance in BOO rats was significantly reduced. Further research found that SFN improved bladder compliance by upregulating matrix metalloproteinases-1 (MMP-1) and downregulating tissue inhibitors of metalloproteinases-1 (TIMP-1) expression. At the same time, they also found that SFN could inhibit the decrease in BOO compliance by reducing the collagen I/III ratio.

## 5 Effects on bladder cancer

### 5.1 Induction of apoptosis in bladder cancer cells

Apoptosis is one of the most famous active forms of programmed cell death, and it plays a key role in limiting cell population expansion, tumor cell death, and maintaining tissue homeostasis. There are three main apoptotic pathways: the endogenous mitochondrial pathway, the exogenous death receptor pathway, and the endoplasmic reticulum (ER) pathway (Gupta and Gollapudi, 2007). Among these, the Bcl-2 protein family is an important regulator of cell survival and apoptosis, while cysteine-containing cysteinases are key enzymes in the initiation and execution of apoptosis (Czabotar et al., 2014; Boice and Bouchier-Hayes, 2020). Available studies have shown that SFN can induce BC apoptosis through all three of these pathways. Studies have shown that survivin is an important anti-apoptotic protein that is associated with poor prognosis in BC and is also a predictor of BC disease progression (Rosenblatt et al., 2008; Jeon et al., 2013). Research has confirmed that SFN can significantly increase caspase3/7 activity and poly (ADP ribose) polymerase (PARP) cleavage while reducing the expression of survivin protein in BC cells (Abbaoui et al., 2012; Wang and Shan, 2012). At the same time, SFN also reduces the expression of the tyrosine kinase receptors EGFR and HER2/neu in aggressive BC cells (Abbaoui et al., 2012). In addition, Nrf2, reactive oxygen species (ROS), and mitochondria play an important role in SFN-induced apoptosis. SFN alone or in combination can increase the activation and expression of caspase-3, caspase-8, caspase-9, and PARP cleavage in BC cells (Tang and Zhang, 2005; Jo et al., 2014; Park et al., 2014; Jin et al., 2018). However, SFN can also induce ROS production and induce MMP depolarization to disrupt mitochondrial membrane integrity, thereby inducing apoptosis (Jo et al., 2014; Park et al., 2014; Jin et al., 2018). Notably, tumor necrosis factor-related apoptosis-inducing ligand (TRAIL) induces apoptosis in a variety of cancer cells by binding to the death receptors DR4 and DR5. SFN/TRAIL upregulates DR5 expression and ROS production by inhibiting Nrf2 activation. Thereby promoting apoptosis in TRAIL-resistant BC cells (Jin et al., 2018). Secondly, SFN upregulates ROS production, induces mitochondrial oxidative damage, mitochondrial membrane potential depolarization, cytochrome c release, and induces an imbalance between Bax and Bcl-2; downregulates the proteins of inhibitor of apoptosis proteins (IAP) family members, activates caspase-9, caspase −3, and cleavage of PARP; upregulates glucose-regulated protein (GRP) 78 and C/EBP-homologous protein (CHOP) expression; and accumulation of p-Nrf2 in the nucleus; thereby induces apoptosis in BC cells. However, the regulatory effects of SFN on these proteins may be dependent on the ER and Nrf2-ARE pathways (Jin et al., 2018).

### 5.2 Induction of bladder cancer cells cycle arrest

The process of cell cycle progression is strictly dependent on the regulation of cyclins, cell cycle-dependent protein kinases (CDK), and CDK inhibitors (CKI), which ultimately complete DNA replication and cell proliferation. Abnormal expression of cell cycle proteins can cause abnormal cell proliferation, eventually leading to tumor development (Dang et al., 2021). It is worth noting that there are three major cell cycle checkpoints that regulate the cell cycle process, including the G1/S checkpoint (restriction point), the G2/M DNA damage checkpoint, and the spindle assembly checkpoint (Barnum and O'Connell, 2014; Satyanarayana and Kaldis, 2009; Gao et al., 2020). Therefore, targeting the cell cycle checkpoints and disrupting the cell cycle is an important direction for treating cancer. Increasing evidence suggests that SFN can significantly induce G2/M cell cycle arrest in BC cells, thereby inhibiting their proliferation (Shan et al., 2006; Abbaoui et al., 2012; Jin et al., 2018; Xie et al., 2022). Part of the mechanism is due to the upregulation of CDK1, CDK2, cyclin A, and cyclin B by SFN, which alters the CDK-cyclin axis (Xie et al., 2022). Interestingly, histone H3 phosphorylation, a mid-phase marker of mitosis, regulates transcriptional activity in G1 phase and affects chromatin condensation in G2/M phase, and phosphorylation modification of histone H3 serine 10 [H3(Ser10)] is closely related to cell cycle and gene transcriptional regulation (Nowak and Corces, 2004). Previous studies have shown that SFN can significantly increase the levels of cyclin B1, Cdk1, the cyclin B1/Cdk1 complex, and phosphorylated histone H3 (Ser 10) in BC cells, inducing mitotic arrest. However, the mechanism may be mediated by SFN-dependent activation of ROS-dependent cyclin B1/Cdk1 complexes and histone H3 (Ser 10) phosphorylation (Park et al., 2014). In addition, SFN can also block the cell cycle by regulating CKI. p27, a very important CKI, inhibits cell proliferation by binding to cyclin/CDK complexes (Razavipour et al., 2020). Interestingly, SFN can block the G0/G1 checkpoint, thereby inhibiting breast cancer cell proliferation. However, this mechanism seems to be significantly associated with upregulation of p27 (Shan et al., 2006).

### 5.3 Inhibition of growth, invasion and metastasis of bladder cancer cells

Tumor cell growth, migration, and invasion are dynamic and complex processes that contribute to the progression of many diseases. The ability of tumor cells to migrate and invade allows them to change locations within tissues and detach from primary tumors, resulting in disease spread. It also allows tumor cells to enter lymph and blood vessels to spread to distant organs and establish metastases (Duff and Long, 2017). Fortunately, current literature reports have shown that SFN can significantly inhibit the growth, invasion, and metastasis of BC cells through multiple pathways (Tang et al., 2006; Zhang et al., 2006; Wang and Shan, 2012; Bao et al., 2014). The PI3K/Akt/mTOR pathway is involved in many cellular processes, including motility, growth, metabolism, and angiogenesis, and its abnormal activation often promotes BC growth and drug resistance (Shaw and Cantley, 2006; Courtney et al., 2010; Xie et al., 2022). Studies have shown that low doses of SFN promote BC angiogenesis, while high doses significantly inhibit it (Bao et al., 2014). In addition, SFN can reduce the levels of AKT, mTOR, mTOR complex 1 (Raptor), and mTOR complex 2 (Rictor) in most BC cells, thereby inhibiting their growth. This seems to be partially due to the inhibition of the PI3K/Akt/mTOR pathway. Interestingly, increased levels of pAkt and pRictor have been observed in a small subset of BC cells, but their relevance remains unclear (Xie et al., 2022). Additionally, FAT atypical cadherin 1 (FAT1) is highly expressed in breast cancer tissues or cells and is associated with a poor prognosis. SFN can dose-dependently reduce FAT1 expression to inhibit the vitality, invasion, and metastasis of BC cells (Wang et al., 2020). The epithelial-to-mesenchymal transition (EMT) is the biological process by which epithelial cells acquire a mesenchymal phenotype, which is mediated by EMT transcription factors (TFs), including SNAIL (Snail1/Snail and SNAIL2/Slug), TWIST (Twist1 and Twist2), and zinc-finger E-box-binding (ZEB). In addition to regulating each other, TF also leads to epithelial gene repression (such as downregulation of E-cadherin, ZO-1, and occludin) and mesenchymal gene induction (such as upregulation of N-cadherin, vimentin, and fibronectin) (Lamouille et al., 2014; Debnath et al., 2022). Notably, SFN has been shown to clearly regulate EMT processes in BC cells. Previous studies have shown that upon treatment of human BC cells with SFN, on the one hand, SFN dose-dependently downregulates cyclooxygenase-2 (COX2), MMP-2, and MMP-9; on the other hand, SFN induces E-cadherin expression through inhibition of Snail and ZEB1. Interestingly, MiR200c is a key regulator of EMT in BC cells, and SFN can also inhibit ZEB1 and induce E-cadherin expression by upregulating MiR200c. These are mechanisms by which SFN inhibits the EMT process in BC cells through the COX-2/MMPs/ZEB1, Snail and miR-200c/ZEB1 pathways (Shan et al., 2013). Secondly, histone H1 is closely associated with BC development and progression, with common modifications such as acetylation, phosphorylation, methylation, ubiquitination, SUMOlyation, and ADP-ribosylation, where the histone acetylation state is regulated by histone acetyltransferase (HAT) and histone deacetylase (HDAC) (Portela and Esteller, 2010; Telu et al., 2013; Shen et al., 2015). Excitingly, SFN reduces histone H1 phosphorylation by inhibiting HDACs 1, 2, 4, 6 and HATs and enhancing the activity of the phosphatases PP1β and PP2AD (Abbaoui et al., 2017).

### 5.4 Inhibition of glucose metabolism in bladder cancer cells

**FIGURE 4:**
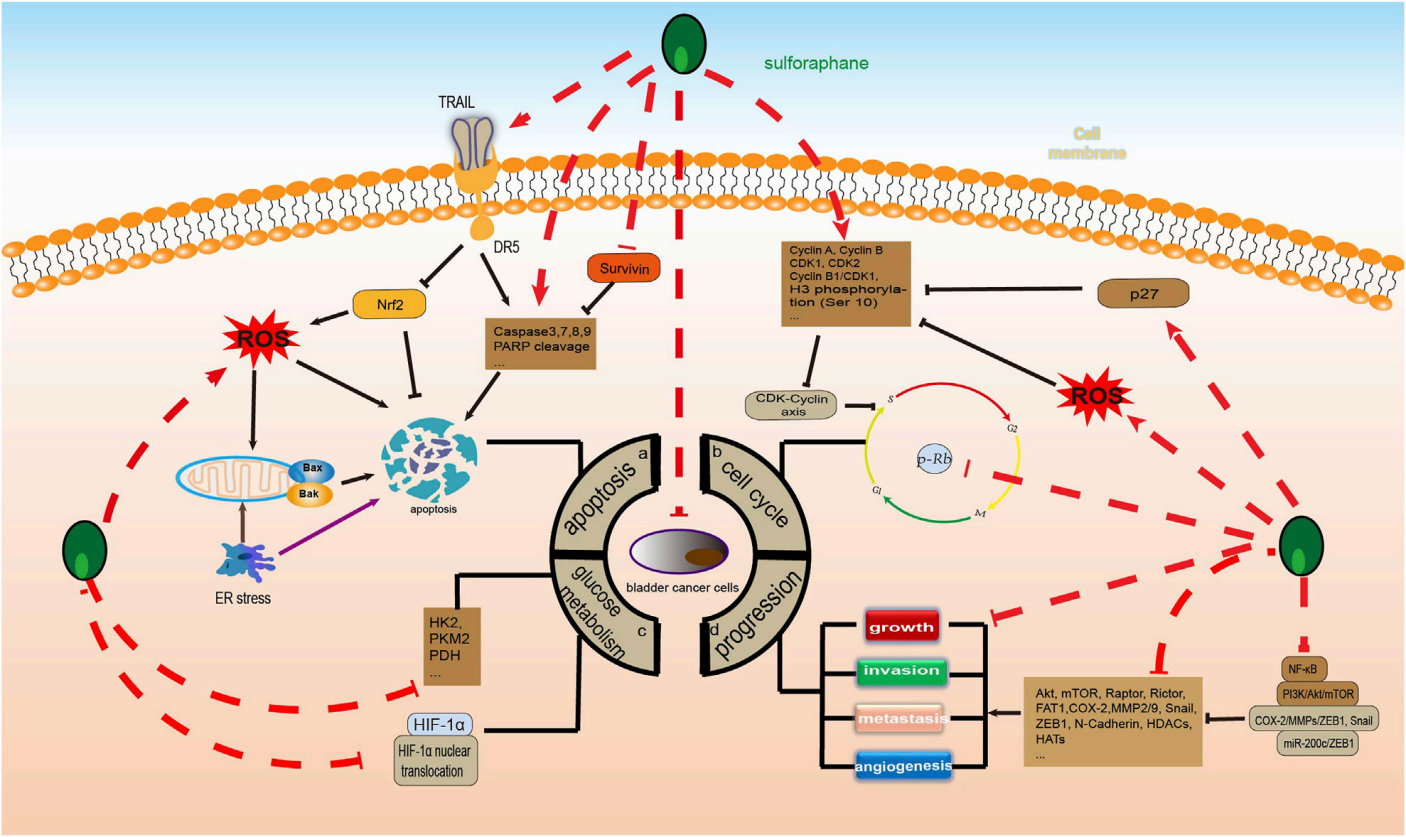
Sulforaphane inhibits the molecular process of bladder cancer (induced by sulforaphane are noted by using →, while the inhibition represented by ⊣ symbol). The occurrence of bladder cancer is closely related to (A) apoptosis, (B) cell cycle, (C) glucose metabolism and (D) progression.

As we all know, tumor cell glucose metabolism is closely related to the occurrence, progression, invasion, metastasis, and treatment of diseases. Tumor cells often achieve sustained growth by reprogramming their glucose metabolism. Even under conditions of sufficient oxygen, cancer cells can still generate adenosine triphosphate (ATP) and lactate by high-speed glycolysis, a phenomenon known as the Warburg effect (WARBURG, 1956). The production of a large amount of ATP and raw materials through the Warburg effect contributes to the proliferation and progression of tumor cells. Therefore, inhibiting glucose metabolism reprogramming is of great significance in BC and even other malignant tumors. According to existing research, SFN can inhibit glucose metabolism in human liver cancer, gastric cancer, and prostate cancer cells by downregulating the expression of serine palmitoyltransferase 3 (SPTLC3), glycolysis-related enzymes including hexokinase 2 (HK2), pyruvate kinase M2 (PKM2), lactate dehydrogenase A (LDHA), and T-box transcription factor 15 (TBX15), and upregulating the expression of kinesin family member 2 C (KIF2C). These mechanisms are partially due to activation of the insulin receptor substrate 1 (IRS-1)/Akt pathway and the TBX15/KIF2C pathway (Singh et al., 2019; Teng et al., 2019; Gu and Wu, 2023). Therefore, SFN exhibits great potential for anti-tumor glucose metabolism. Research has shown that although the systemic metabolic spectrum of BC is still unclear, there is a severe glucose metabolism disorder in its urine (Putluri et al., 2011), serum (Bansal et al., 2013), and cell lines (Dettmer et al., 2013). Fortunately, SFN has shown significant inhibitory effects on BC glucose metabolism. According to reports, SFN can significantly reduce a variety of glucose metabolism enzymes, including HK2, PKM2, and pyruvate dehydrogenase (PDH), to downregulate the glycolysis of BC cells and inhibit their proliferation in in vivo and *in vitro* experiments. However, this seems to be related to blocking the AKT1/HK2 axis (Huang et al., 2022). In addition, SFN significantly inhibits the proliferation of BC cells under hypoxic conditions compared to normoxic conditions. SFN can lower the glycolysis metabolism in the hypoxic microenvironment by downregulating hypoxia-inducible factor-1 alpha (HIF-1α) induced by hypoxia and blocking HIF-1α nuclear translocation in NMIBC cell lines, thereby inhibiting the proliferation of NMIBC cells (Xia et al., 2019) (Figure 4).

### 5.5 Therapeutic effect of sulforaphane combined with drugs/carcinogens

As new anticancer drugs continue to emerge, chemotherapy resistance in traditional cancer treatment regimens, as well as severe side effects under conventional treatment, have prompted many cancer patients to choose complementary or alternative medicine (CAM) treatment regimens. In fact, the combination of traditional Chinese and Western medicine, especially green chemoprevention, has become a very popular option, especially for late-stage tumors and patients with poor prognosis under conventional treatment (Irmak et al., 2019; Schuerger et al., 2019). The combination of these natural compounds and conventional chemotherapy drugs increases the toxicity of cancer cells through synergistic effects, reducing treatment doses and toxicity (Singh et al., 2016; Zhou et al., 2016). It is worth mentioning that SFN has become an important choice for BC chemoprophylaxis and new drug development. Due to its low toxicity, it has been studied jointly with many chemotherapy drugs or carcinogens and has shown significant preclinical BC prevention effects.

In fact, the long-term use of the mTOR inhibitor Everolimus to inhibit tumor growth and spread has been unsuccessful due to resistance caused by genomic instability induced by long-term treatment with Everolimus, but the underlying mechanisms are currently unclear (Burrell and Swanton, 2014). It has been reported that SFN can inhibit resistance-related tumor dissemination during treatment of BC with everolimus, demonstrating great potential for treating BC patients who are resistant to mTOR inhibitors. Studies have shown that after treatment with 0.5 nM everolimus, or 2.5 μM SFN, or 0.5 nM everolimus +2.5 μM SFN for 8 weeks, everolimus enhanced the chemotactic movement of RT112 cells, while SFN or the SFN-Everolimus combination significantly inhibited the chemotactic movement of RT112 cells. The mechanism seems to be that the SFN or SFN-Everolimus combination inhibits the mTOR complex pRictor and regulates CD44 receptor variants (upregulates CD44v4 and CD44v7) and integrin α and β subtypes (upregulates α6, αV and β1, and downregulates β4) (Justin et al., 2020a; Justin et al., 2020b). In addition, the combination of the carbonic anhydrase (CA) inhibitor acetazolamide and SFN treatment can significantly inhibit the survival of BC cells. Acetazolamide combined with SFN treatment can significantly inhibit BC growth *in vivo* and *in vitro*, produce effective anti-proliferative and anti-cloning effects, induce cell apoptosis through activation of caspase-3 and PARP, and inhibit the EMT process of BC cells by downregulating the levels of CA9, E-cadherin, N-cadherin, and vimentin. However, the reduction of these components may be due to downregulation of the survival-mediated Akt pathway (Islam et al., 2016a). Furthermore, the use of SFN in combination with cisplatin, docetaxel, and chimeric antigen receptor-modified T (CAR-T) cell therapy has also shown good therapeutic effects, even helping to alleviate some toxic side effects of certain chemotherapy drugs (Kerr et al., 2018; Calcabrini et al., 2020; Shen et al., 2021).

Furthermore, the main bladder carcinogen is 4-aminobiphenyl (ABP), and its induction of high levels of ABP-DNA adducts is associated with more aggressive tumor behavior, with 80% of ABP-DNA adducts being dG-C8-ABP (Block et al., 1978; Talaska et al., 1991; Bookland et al., 1992). Research has shown that DING et al. found that ABP can induce BC cell DNA damage in a dose-dependent manner through measuring dG-C8-ABP, while SFN can significantly reduce the levels of dG-C8-ABP in both *in vivo* and *in vitro* experiments, thus inhibiting ABP-induced bladder DNA damage. However, its inhibitory effect appears to be related to the activation of the Nrf2 signaling pathway, but the molecular mechanism by which Nrf2 inhibits ABP-induced DNA damage is not known, nor can the exact Nrf2 regulatory gene that mediates the anti-ABP activity of SFN be identified (Ding et al., 2010). Secondly, N-butyl-N-(4-hydroxybutyl) nitrosamine (BBN) is the most commonly used carcinogen in bladder cancer research, and its carcinogenicity is limited to the bladder (He et al., 2012). It has been identified as an effective and specific bladder carcinogen in rat studies (Oliveira et al., 2006). Fortunately, in a C57BL/6 mouse bladder cancer model induced by BBN with or without SFN treatment for 23 weeks, SFN significantly improved the abnormal fecal microbiota composition, intestinal epithelial barrier disruption, and inflammatory response of BC mice induced by BBN. However, the mechanism includes normalization of gut microbiota imbalance, increased fecal butyric acid levels, and expression of tight junction proteins, G protein-coupled receptor 41 (GPR41), and glucagon-like peptide 2 (GLP2) to improve intestinal mucosal damage, and reduce the levels of cytokines (IL-6) and secretory immunoglobulin A (SIgA) to reduce inflammation and immune response (He et al., 2018).

## 6 Network pharmacological analysis

**FIGURE 5:**
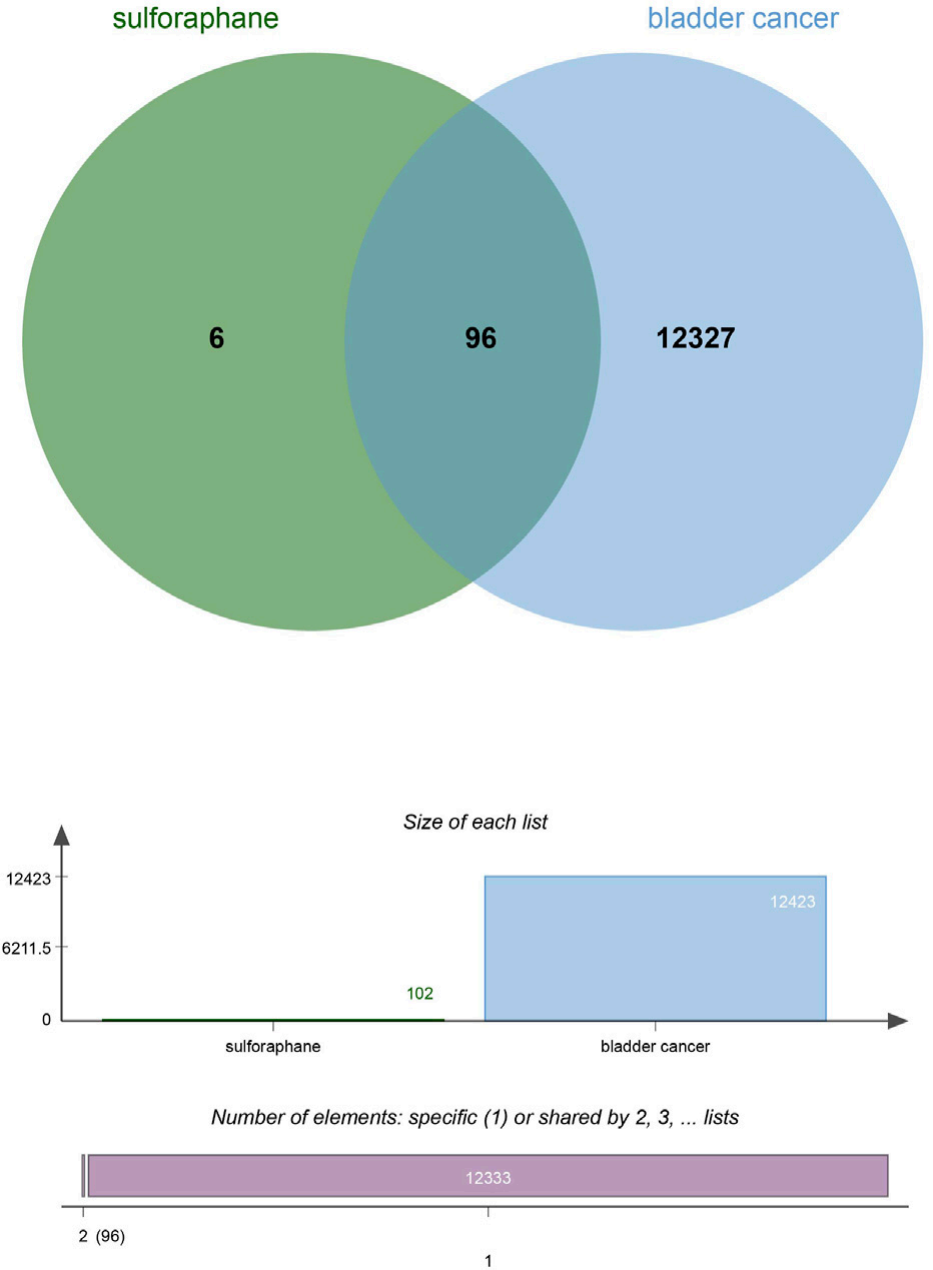
Venny of sulforaphane and bladder cancer.

To explore and validate the target and molecular mechanisms of SFN on BC, we conducted a network pharmacology analysis of SFN and BC. First, we identified the structure of sulforaphane through Pubchem (https://pubchem.ncbi.nlm.nih.gov), and used the Swiss Target Prediction database (http://www.swisstargetprediction.ch/) and the Traditional Chinese Medicine Systems Pharmacology database (https://www.tcmsp-e.com/) to screen drug targets. We then submitted the collected targets to the UniProt database (https://www.uniprot.org/), with the species limited to “*Homo sapiens*”. We converted the protein targets to official gene names and selected gene targets with probabilities greater than 0 in the Swiss Target Prediction database, excluding duplicate genes, resulting in drug targets for sulforaphane (102). Next, we searched Genecards (https://www.genecards.org/), OMIM (https://www.omim.org/), and Disgenet databases (https://www.disgenet.org/) using “bladder cancer” as a keyword to obtain disease targets. After removing duplicate targets from the three databases, we obtained 12,423 disease target genes. Finally, we input the drug target genes and disease target genes obtained using the above methods into the online Venny 2.1 plot platform (https://www.bioinformatics.com.cn/) to obtain the intersectional target genes of “bladder cancer” and “sulforaphane (102)” (Figure 5).

**FIGURE 6:**
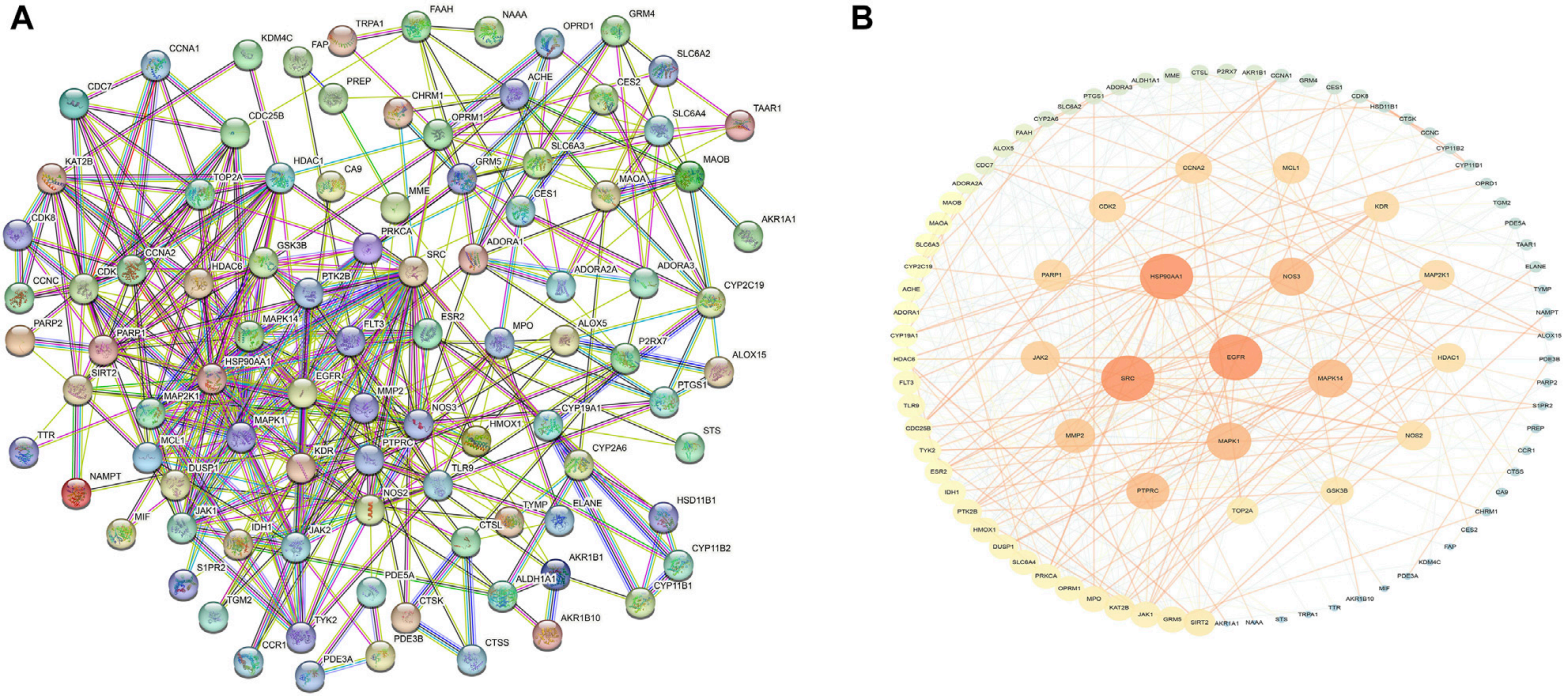
Protein network of sulforaphane and bladder cancer. **(A)** Protein interaction network, **(B)**Protein network analysis.

**FIGURE 7:**
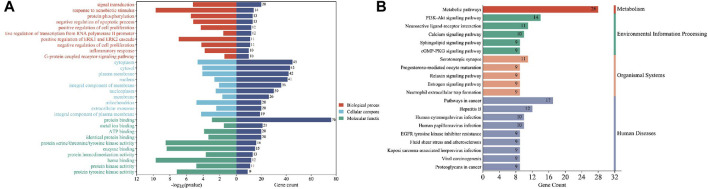
Enrichment analysis of sulforaphane and bladder cancer. **(A)** GO enrichment analysis, **(B)** KEGG enrichment analysis.

These cross-genetic targets are considered potential targets for SFN therapy in BC, and we performed an analysis on them through a series of methods. First, we uploaded these genes to the String online database (https://string-db.org/), generating a protein-protein interaction network map. We set the species to “human” and integrated the comprehensive score >0.4 as the critical value for inclusion in the network. Then, we used Cytoscape 3.9.1 to exclude irrelevant gene targets and further visualize these results (Figure 6), identifying key targets of sulforaphane. Additionally, we analyzed the function of the Gene Ontology (GO) and the Kyoto Encyclopedia of Genes and Genomes (KEGG) pathways. We input these gene data into the David data platform (https://david.ncifcrf.gov/tools.jsp) and set the species as “Homo species,” further analyzing the enrichment analysis of SFN in BC-related biological processes (BP), cellular components (CC), molecular functions (MF) and signal pathways. For the obtained information, we meet the value < 0.05 requirement and selected the top 10 enrichment information for BP, CC, and MF in sequence according to gene number, as well as the top 20 enrichment information for KEGG, and visually analyzed the results using a bioinformatics online platform (https://www.bioinformatics.com.cn/) (Figure 7).

Finally, our results indicate that SFN has very high targeting activity toward BC. Among them, the heat shock protein 90 alpha family class A member 1 (HSP90AA1), proto-oncogene tyrosine-protein kinase SRC (SRC), epidermal growth factor receptor (EGFR), MAPK1, MAPK14, nitric oxide synthase 3 (NOS3), and protein tyrosine phosphatase receptor type C (PTPRC) play key roles in the anti-BC process of SFN. In addition, the cellular components of the GO enrichment analysis indicated that the targets of SFN interfered with the normal assembly of various cellular components, including the cytoplasm, cytosol, plasma membrane, nucleus, and mitochondria. This further indicates that the workplace of SFN is within the cell. Furthermore, the molecular functions and biological processes of GO enrichment analysis suggest that the targets of SFN are involved in the activation and binding of a range of cellular receptors and cascade downstream signaling pathways, as well as the regulation of cell proliferation and apoptosis, and modulate the activity of a number of protein kinases and the production of ATP. In addition, KEGG enrichment analysis showed that metabolic pathways, the PI3K/Akt signaling pathway, the Calcium signaling pathway, and the cGMP-PKG signaling pathway play key roles in the anti-BC process of SFN. These pathways play important roles in the growth, invasion, metastasis, angiogenesis, and energy metabolism of BC. Taken together, our network analysis and available laboratory data suggest that SFN can inhibit BC metastasis, invasion, and progression through multiple targets and pathways.

## 7 Molecular mechanism and targets of sulforaphane against bladder cancer

As we all know, the occurrence and development of BC involve multiple pathways and targets, and changes in the activity of these signaling pathways significantly affect many cell activities, from growth and proliferation to apoptosis, invasion, and metastasis. Previous studies have shown that the occurrence of BC is not only the result of the involvement of multiple oncogenes (ras, p53, RB1, FGFR3, EGFR), but is also related to the abnormal activation of multiple signaling pathways (PI3K/Akt, Wnt/β-catenin, JAK/STAT, Notch, NF-κB, MAPK, Hedgehog) (Zhang et al., 2015; Chestnut et al., 2021). Through network pharmacology analysis, GO enrichment analysis, KEGG enrichment analysis, and a review of a large number of previous studies, we found that SFN has extremely high targeting activity against these targets and pathways. It is worth mentioning that because SFN can inhibit the progression of BC cells through multiple targets and pathways, both domestic and foreign scholars are currently conducting extensive research on its molecular mechanism.

Mitogen-activated protein kinase (MAPK) is a type of serine/threonine protein kinase that is a key signaling pathway regulating various cell processes, including cell proliferation, differentiation, apoptosis, angiogenesis, migration, invasion, etc. Notably, MAPK, including extracellular signal-regulated kinase (ERK), c-Jun NH(2)-terminal kinase (JNK), and p38 MAPK, are associated with bladder cell carcinogenesis and bladder tumor progression (Hepburn et al., 2012; Cancer Genome Atlas Research Network, 2014; Lv et al., 2017; Deramaudt et al., 2020). Fortunately, SFN can not only directly reduce the expression and phosphorylation of MAPK but also significantly inhibit the activation and expression of MAPK signaling pathway-related target proteins, such as ERK, JNK, and p38 MAPK, etc. (Subedi et al., 2019; Yang et al., 2022). In addition, SFN can not only inhibit the expression of MAPK-related molecules (Erk1/2, JNK, and p38 MAPK) by activating Nrf2 expression but also by increasing ROS production, thereby inhibiting the MAPK/activator protein 1 (AP-1) signaling pathway (Chaiprasongsuk et al., 2017; Li S. et al., 2022). So far, p38 has become a target for a series of anti-cancer drugs (Yong et al., 2009). The activation of p38 MAPK plays an anti-BC role through various pathways, such as blocking the cell cycle process (Yang et al., 2021), inducing cell apoptosis (Zhang et al., 2014; Chen et al., 2022), inhibiting cell invasion and metastasis (Kumar et al., 2010; Piao et al., 2021), and through the antioxidant response element (ARE) driving genes and COX-2 (Shan et al., 2009; Shan et al., 2010). It is worth mentioning that SFN can significantly upregulate the expression of Nrf2-dependent enzymes (glutathione transferase and thioredoxin reductase) and downregulate the expression of COX-2, but p38 MAPK inhibitors can reverse this effect. The mechanism seems to be that SFN activates p38 MAPK, leading to the activation of Nrf2 mediated by p38 MAPK (Shan et al., 2010).

**FIGURE 8:**
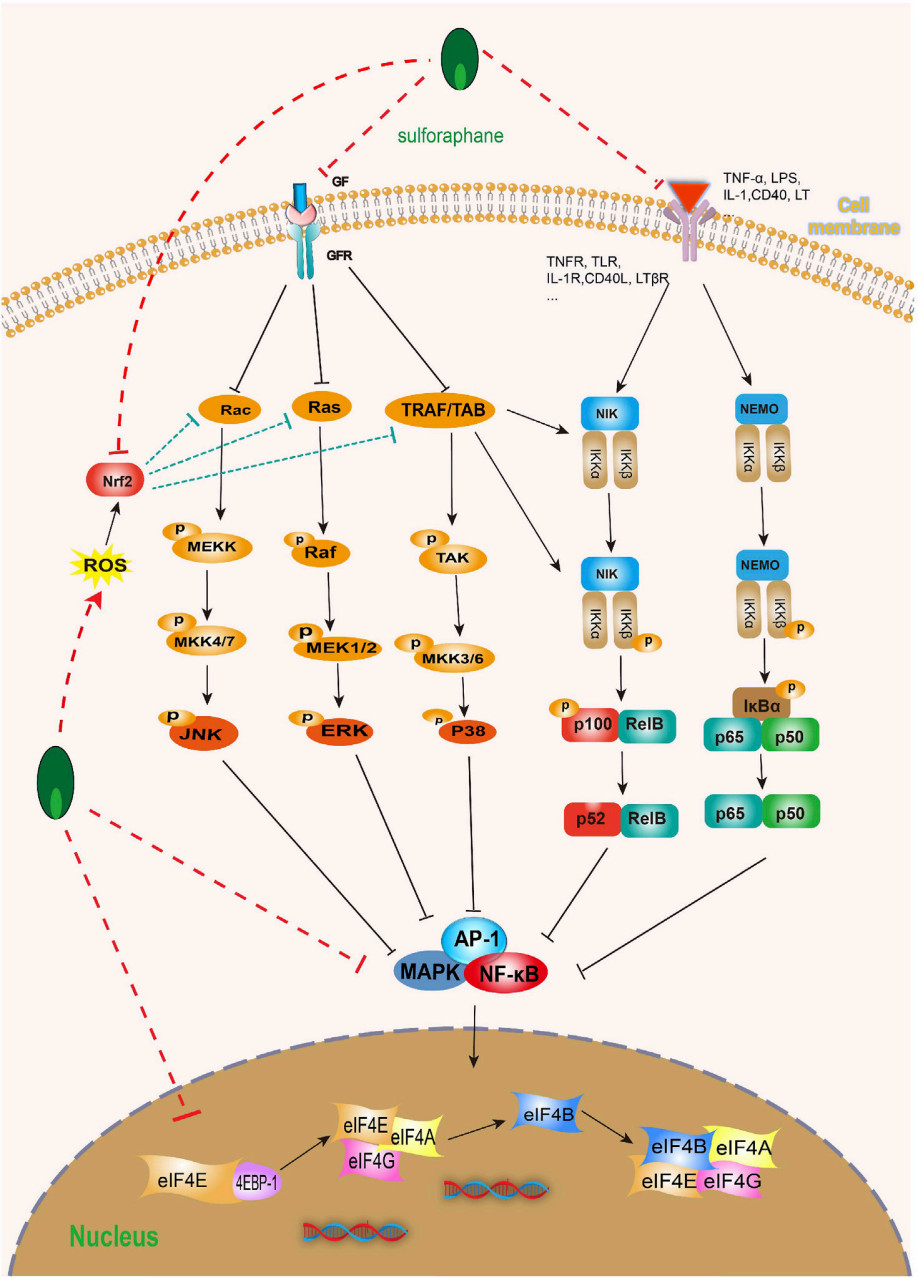
Effect of sulforaphane on MAPK and NF-κB (induced by sulforaphane are noted by using →, while the inhibition represented by ⊣ symbol). MAPK signal pathway is correlated with NF-κB. Sulforaphane can block the occurrence, development, invasion and migration of bladder cancer cells by inhibiting common upstream and downstream molecules, multiple receptors and ligands.

As we all know, the nuclear factor kappa-B (NF-κB) family is a key regulatory factor for cell survival, and its NF-κB signaling participates in many biological processes, including immune and inflammatory responses, proliferation, apoptosis, and EMT (Liu et al., 2017; Zhang et al., 2017). However, the key step in activating typical NF-κB is the phosphorylation-dependent activation of the IκB kinases (IKKs) complex (Hayden and Ghosh, 2008; Israël, 2010). It is worth noting that some evidence suggests that the abnormal upregulation of NF-κB transcription factors is closely related to poor prognosis in BC patients (Doyle and O'Neill, 2006; Perkins and Gilmore, 2006; Chiang et al., 2019; Walter et al., 2020). In fact, more and more evidence suggests that SFN can inhibit the abnormal activation of NF-κB in various ways. SFN can not only inhibit IκB degradation and phosphorylation (Cancer Genome Atlas Research Network, 2014; Song et al., 2022; Yang et al., 2022), but also inhibit the NF-κB signaling pathway by activating NF-κB nuclear translocation (Jeong et al., 2010; Subedi et al., 2019). Furthermore, a large amount of evidence indicates that COX-2 is overexpressed in human BC and BC animal models, and it is closely related to the progression, prognosis, and recurrence of BC (Shirahama et al., 2001; Klein et al., 2005), and NF-κB is closely related to the expression of COX-2 mRNA (Garg and Aggarwal, 2002). It is worth mentioning that SFN can time and dose-dependently activate NF-κB nuclear translocation in T24 BC cells and inhibit NF-κB DNA binding to the COX-2 promoter, thereby inhibiting COX-2 mRNA and protein levels (Shan et al., 2009) (Figure 8).

## 8 Effect of sulforaphane on bladder cancer stem cells

Cancer stem cells (CSCs) are tumor-initiating clonogenic cells capable of maintaining cellular heterogeneity, self-renewal, and differentiation, persisting in the tumor microenvironment long-term, and playing a role in tumor growth, metastasis, chemoresistance, and recurrence (Zhang et al., 2012; Bellmunt, 2018). However, bladder CSCs (BCSCs) represent a distinct type of CSCs originating from bladder epithelial stem cells and non-stem cells with autophagy, sphere-forming, and multilineage differentiation capabilities, which was first identified in urothelial carcinoma of the bladder in 2009 (Ohishi et al., 2015; Yang et al., 2017). The stemness of BCSCs is regulated by various targets and pathways, making them an ideal target for intervention therapy in BC. Notably, BCSCs exhibit high heterogeneity, with the stem cell populations and characteristics varying significantly among different subtypes of BC. CD44, CK5, P-cadherin, and CK14 are prominently expressed in CSCs derived from NMIBC, while additional stem cell markers, such as ALDH, Nestin, CD133, CD90, NANOG, OCT4, and SOX, are expressed in CSCs derived from MIBC, which are partially associated with invasiveness, chemoresistance, and self-renewal in MIBC (Ooki et al., 2018; Hayashi et al., 2020). Therefore, targeting BCSCs is a promising treatment strategy for treating BC and controlling recurrence and metastasis.

Excitingly, SFN has demonstrated significant value in anti BCSCs. Previous studies have shown that ΔNp63α and TAp63α, two major subtypes of the p63 family of proteins, play essential roles in stemness maintenance and cell proliferation (Yang et al., 1998; Melino et al., 2015). Importantly, SFN has been found to dose-dependently reduce the expression levels of ΔNp63α, TAp63α, and stem cell markers such as NANOG, OCT4, and SOX2, as well as diminish the sphere-forming ability of CSCs, which can be reversed by overexpression of ΔNp63α and TAp63α (Chen et al., 2020; Chen et al., 2022a). This mechanism appears to be partially mediated by SFN inhibiting the ΔNp63α/NANOG/OCT4/SOX2 axis (Chen et al., 2022a). Additionally, ZO-1, an epithelial-mesenchymal marker that is associated with EMT and CSCs characteristics in BC (Islam et al., 2016b). SFN has been shown to promote the expression of ZO-1 to reduce the expression of stem cell markers (CD133, CD44, NANOG, and OCT4) and nuclear expression of β-catenin in CSCs. The mechanism involves SFN modulating the ZO-1/β-catenin axis to suppress CSCs stemness (Chen et al., 2022b). Furthermore, the Sonic Hedgehog (SHH) signaling pathway also plays an important role in the characteristics of BCSCs, which regulate self-renewal, proliferation, and invasiveness (Islam et al., 2016b). Luckily, SFN significantly lowers the expression of key components of the SHH pathway (Shh, Smo, and Gli1) and inhibits tumor sphere formation, thereby suppressing the stemness of cancer cells (Ge et al., 2019; Wang et al., 2021). These findings suggest that SFN can inhibit BCSCs self-renewal by targeting the SHH signaling pathway. Moreover, previous research has shown that the combination of acetazolamide (AZ) and SFN significantly inhibits BC growth, progression, and EMT processes (Islam et al., 2016a). Recent studies have revealed that the combination of AZ and SFN can also significantly reduce the sphere-forming ability and expression of CSCs markers such as Oct-4, Sox-2, and Nanog, thus suppressing cancer cell stemness (Bayat Mokhtari et al., 2019). Additionally, miR-124, one of the most extensively studied microRNAs, has been demonstrated to inhibit BC cell proliferation through targeting CDK4 (Cao et al., 2019). Interestingly, SFN suppresses the expression of stem cell markers (CD44 and EpCAM), IL-6R, STAT3, and p-STAT3 in a dose-dependent manner on the one hand, and on the other hand, significantly increases the expression of miR-124 in a dose-dependent manner. However, knockdown of miR-124 eliminates the effect of SFN on CSC-like characteristics in further experiments (Wang et al., 2016). These results indicate that SFN can target BCSCs through the miR-124/IL-6R/STAT3 axis.

## 9 The dilemma of clinical application of sulforaphane

Although SFN was isolated and identified in 1959, it was not until 1992 that it gained attention. Despite increasing research on SFN, the field remains small, with the main challenge being the definition and optimization of plant formulations, including procurement, composition, elucidation of key pharmacokinetic parameters, and dose selection (Palliyaguru et al., 2018). In fact, SFN is unstable in aqueous solutions and at high temperatures, sensitive to oxygen, heat, and alkaline conditions, with a decrease in quantity of 20% after cooking, 36% after frying, and 88% after boiling (Beevi et al., 2010; Baenas et al., 2019). Therefore, SFN is difficult to deal with in the pharmaceutical process, greatly reducing its success rate after oral administration. Moreover, the effective dose and lethal dose range of SFN have not been determined. In animal models, the dose range of sulforaphane is 5–100 mg/kg for reducing tumors (Singh et al., 2019; Kaiser et al., 2021). For a person weighing 60 kg, this is equivalent to 300–6000 mg, which clearly exceeds their threshold. In addition, in some clinical trials, the test dose of SFN cannot be accurately converted to the amount of vegetables consumed. Research has shown that the average concentration of SFN in raw broccoli is 0.38 μmol/g (Yagishita et al., 2019). However, most of the doses of SFN used in clinical trials range from 25 to 800 μmol, equivalent to about 65–2,105 g of raw broccoli, which is actually difficult to consume. In fact, high concentrations of SFN have been shown to exhibit significant toxicity toward normal cells. *In vitro* studies have shown clear adverse effects with SFN at concentrations of 10–30 μM, including induction of DNA, RNA, and mitochondrial damage (Zhang et al., 2005; Sestili et al., 2010; Fahey et al., 2015). Secondly, how to improve the bioavailability of SFN is also a key issue. It has been reported that the ability of individuals to use gut myrosinase to convert glucoraphanin into SFN varies widely (Fimognari et al., 2012; Sivapalan et al., 2018). However, even if the same SFN and myrosinase are given to subjects at the same time, there is still variability in the conversion and bioavailability of SFN among individuals (Shapiro et al., 2001). In addition, there is also concern about mitigating the toxicity of SFN. SFN has a low toxicity, and most *in vitro* and *in vivo* experiments have been conducted at concentrations ranging from 0 to 40 μM without significant observed toxicity. In human trials, SFN has been relatively safe at low doses with no adverse reactions, and minimal harm has been observed at high doses (Shapiro et al., 2006). Furthermore, although the US Food and Drug Administration has restricted some clinical trials to SFN doses of 200 μmol, the adverse reactions are still negligible, with only one case of grade 2 constipation reported. Moreover, the study suggests that higher doses of SFN may have greater benefits, but further testing is needed (Alumkal et al., 2015). Therefore, the dose-related advantage of SFN in reducing adverse reactions is evident. It can exert positive anti-BC effects by reducing toxic side effects in a dose-dependent manner. Based on this, further testing with more extreme doses is still needed in the future. It is worth mentioning that most of the current research on the impact of SFN on BC is still based on *in vitro* experiments and animal models. Future work needs to validate *in vitro* findings, optimize SFN drug dosage through animal model studies, and conduct more clinical trials for BC patients to improve the bioavailability of SFN. Excitingly, it has been reported that daily oral administration of 200 μM SFN in melanoma patients can achieve plasma levels of 655 ng/mL with good tolerance (Tahata et al., 2018). Drinking 300 mL of broccoli soup per week can lower gene expression in the prostate and a negative correlation between cruciferous vegetable intake and prostate cancer progression has been observed (Traka et al., 2019). In addition, pancreatic ductal adenocarcinoma patients under palliative chemotherapy showed improved outcomes after taking 15 capsules per day (90 mg/508 μmol SFN) for 1 year. However, taking 15 capsules per day can be difficult for some patients, and broccoli sprouts can sometimes exacerbate digestive problems such as constipation, nausea, and vomiting (Lozanovski et al., 2020). This issue also reminds us to develop better-tolerated and more efficient new SFN formulations.

## 10 Conclusion and prospects

As we all know, the occurrence and development of BC involve abnormal regulation of multiple pathways and targets. Therefore, drugs with multiple pathways and targets can play a significant role in the treatment of BC. However, SFN is expected to become an ideal drug for the treatment of BC. SFN is the best anti-cancer active substance found in vegetables and has been widely recognized in recent years. As a natural product, it is cheaper, safer, and easier to obtain than other anti-cancer drugs. In addition, SFN has been shown to have low toxicity, is not oxidizable, and has good tolerance to individual administration, making it an effective natural dietary supplement in many clinical trials. Secondly, our review shows that SFN can inhibit the progression of BC cells through various pathways, including inducing cell apoptosis and cell cycle arrest, inhibiting cell growth, migration, and invasion, regulating cell glucose metabolism, inhibiting tumor angiogenesis, and acting as a synergistic agent for chemotherapy drugs. In addition, it can play an anti-BC and anti-BCSC role by regulating multiple signaling pathways, including PI3K/Akt, NF-kB, MAPK, Nrf2, ZO-1/β-Catenin. miR-124/IL-6R/STAT3, etc.

However, due to the low bioavailability of SFN and its unstable biochemical properties, its clinical application encounters many obstacles. Fortunately, more and more researchers are working to improve its bioavailability and absorption by cancer cells. For example, new SFN injections such as microencapsulation, microspheres, micelles, and nanoparticles are being developed. It is worth mentioning that the current research on the effect of SFN on BC is mostly based on *in vitro* cell experiments and *in vivo* animal experiments. We still need to carefully design more pharmacokinetic and clinical trials to clarify the toxicity, effective dose, and lethal dose range of SFN, which will provide a certain foundation for the development of new drugs. In summary, all research results indicate that SFN is expected to be used as a new or adjunct drug for the treatment of BC.
